# Elucidate biomarkers and the molecular pathways associated with genetic variants that contribute to the etiology of Parkinson’s disease

**DOI:** 10.1007/s13760-025-02897-7

**Published:** 2025-09-30

**Authors:** Hai Duc Nguyen

**Affiliations:** Department of Microbiology, Tulane National Primate Research Center, Louisiana, 70433 USA

**Keywords:** GWAS, Parkinson’s disease, Molecular mechanisms, Biomarkers

## Abstract

Genetic variants can affect signaling pathways that are important in the pathophysiology of Parkinson’s disease (PD). Comprehending their relationship is crucial for the development of diagnostic instruments and preventative drugs for PD. We thoroughly analyzed data from 68 genome-wide association studies to uncover significant genetic variations and clarify the molecular pathways underlying the etiology of Parkinson’s disease (PD) resulting from genetic variants. Six common biomarkers linked to PD were found in all 68 investigations: *SNCA*,* TMEM175*,* BST1*,* RIT2*,* LRRK2*, and *MCCC1*. *SNCA* ($\uparrow$rs5019538 and $\uparrow$rs356182), *LRRK2* ($\uparrow$rs34637584 and $\uparrow$rs76904798), and *SH3GL2* ($\uparrow$rs10756907 and $\downarrow$rs13294100) were the main biomarkers associated with PD. The clinical traits of PD, such as age at onset, cognitive progression, motor progression, composite progression, tremor dominant, and postural instability gait difficulty, have been found to be underpinned by additional biomarkers, including *APOE*,* NTRK2*,* SLCO1B3*,* SLC28A3*,* AQP10*,* SNCAIP*,* ANO2*,* CADM1*,* PTPRD*,* GPR32*,* GPR321*,* SQOR*,* SULT1C2*,* GABRG2*,* CYP4Z1*,* CDH13*, and *FANCF*. Significant evidence was found linking genetic variants linked to an increased risk of PD to reduced dopamine production, receptor recycling, oxidoreductase activity, and increased amyloid-beta accumulation. Considerable evidence links genetic variations with a lower risk of PD due to improved synaptic vesicle signaling, neuron projection development, controlled histone methylation, and excitatory postsynaptic potential. Additionally, we found *MYT1L* and *hsa-miR-20a-5p*, which are essential for understanding the genetic variations linked to PD. These findings provide a solid underpinning for future therapeutic approaches aimed at PD, with a focus on the genetic variants and processes connected to the illness.

**Graphic abstract:**

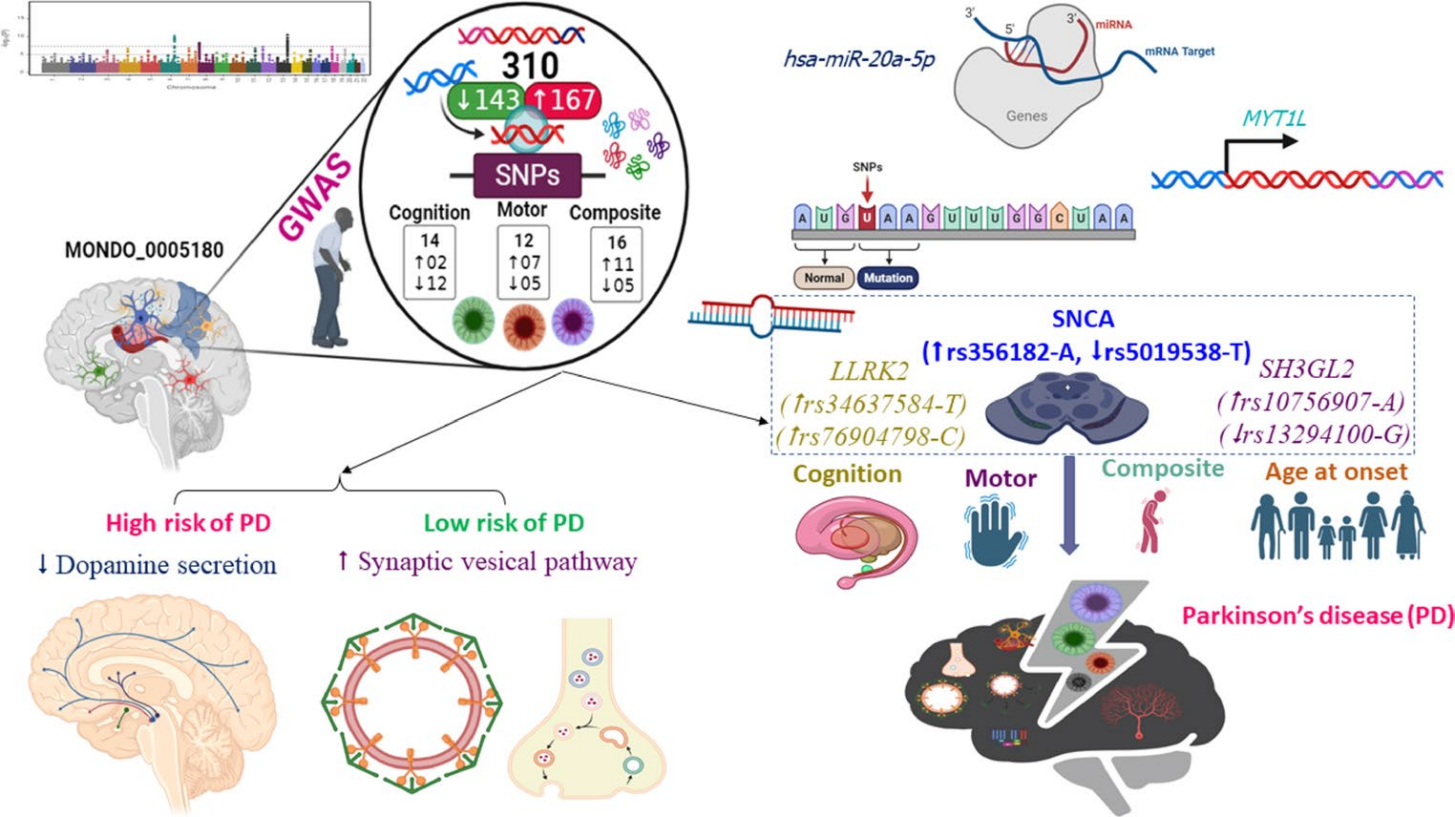

**Supplementary Information:**

The online version contains supplementary material available at 10.1007/s13760-025-02897-7.

## Introduction

Parkinson’s disease (PD) is a prevalent neurological disorder associated with aging. Its hallmark characteristics are the degeneration of dopaminergic neurons and the presence of Lewy bodies in the substantia nigra [18]. PD is characterized by symptoms such as resting tremors, postural instability, bradykinesia, and rigidity. The clinical presentation encompasses non-motor symptoms such as cognitive decline, hyposmia, sleep difficulty, depression, and autonomic dysfunction [8]. These symptoms worsen over time, impairing the patient’s ability to move and overall well-being. The number of PD cases tends to increase in the next few decades, resulting in a significant burden on health systems [18]. Nevertheless, the precise etiology of PD remains uncertain, but genetic factors, the aging process, and environmental factors are pivotal in the development of PD [40].

Over the last decade, around 20 genes have been linked to PD, or parkinsonism, in familial cases [7]. Although detrimental mutations in genes are crucial in the development of PD, only a small number of patients actually possess these mutations [52]. With the development of technology, genome-wide association studies (GWASs) can identify common variations with a small effect size, which have been found to significantly influence the genetic predisposition to PD. Over 300 prevalent variations (e.g., *SNCA*,* LRRK2*,* BST1*,* GCH1*,* VPS13C*,* TMEM175*, and *MAPT*) have been linked to age at onset, progression, and the risk of sporadic PD by GWASs [6, 21, 37, 58]. Although there have been extensive collaborative efforts involving multiple cohorts, the currently identified genetic variations can only account for 22–36% of the heritability of PD in the population of European ancestry [58]. Genetic variations have increased the challenge of finding approaches that can accurately identify, treat, and prevent this condition. Although numerous molecular pathways underlying the pathogenesis of PD associated with these variations have been identified, such as endocytosis, lysosomal function, and mitochondrial function, these molecular pathways seem insufficient to describe the precise effects of genetic variations on PD etiology [45, 58]. Thus, it is imperative to elucidate the precise molecular mechanisms underlying PD pathogenesis associated with genetic variations. Our hypothesis proposes that genetic variations in key genes may impact the biological mechanisms associated with PD. These genetic variations have the capacity to impact signaling pathways that play significant roles in the pathogenesis of PD. Understanding the link between them plays the most important role in the development of diagnostic tools and preventive therapies for PD. Therefore, the objective of this study is to determine the key genetic variations and their corresponding biological pathways that contribute to the etiology of PD using GWAS data from a total of 68 studies.

## Materials and methods

### Determination of putative genetic variants implicated in parkinson’s disease pathogenesis

The present study utilized data on “Parkinson Disease” obtained from the GWAS database (ID: MONDO_0005180) to investigate the potential of genetic variations as biomarkers for predicting and guiding therapy responses in individuals diagnosed with PD. The data was obtained on November 14, 2023. The dataset consists of 561 different genetic variations and risk alleles that were collected from a total of 71 investigations. It includes three symptoms (Parkinson disease, Parkinson’s disease, and paralysis agitans), 29 characteristics (such as Parkinson’s disease, Parkinson disease and Lewy body pathology, Parkinson’s disease (familial), etc.), and two sub-characteristics (secondary Parkinson disease and young adult-onset Parkinsonism). To ensure disease specificity, our analysis exclusively focused on genetic variants associated with PD, excluding entries linked solely to Lewy body pathology or other neurological conditions. In this study, the first stage was excluding a specific number of gene and beta data items that had missing gene (*n* = 34) and beta (*n* = 312) indicators in the specified “mapped genes” and “beta” tabs. We excluded records with missing gene annotations or beta values to ensure data quality and consistency. Although gene inference and beta approximation from odds ratios are possible, these approaches were not considered in this study to avoid potential inaccuracies due to inconsistent annotations and heterogeneous data sources. Afterwards, 8 records were excluded from the study because they had unclear variant and risk allele information. In conclusion, a grand total of 232 records were chosen for further processing (Supplementary Table S1). In order to study the molecular mechanisms behind the development of Parkinson’s disease associated with genetic variations, we specifically selected records that showed the highest beta value and the lowest p-values in 68 studies after cleaning data.

## Sensitivity analysis

To evaluate the impact of excluding 312 records with missing beta values, a sensitivity analysis was conducted. Beta values for a subset of excluded records were estimated using odds ratios (when available) or mean beta values from similar SNPs in the dataset. Re-analysis of hub genes and pathways with these imputed values showed no significant changes in the prioritization of *SNCA*, *LRRK2*, *SH3GL2*, *TMEM175*, *BST1*, *RIT2*, or *MCCC1*, nor in the enrichment of key pathways (e.g., synaptic vesicle endocytosis, dopamine secretion), confirming the robustness of our findings.

## Determining plausible biological pathways linking genetic variants to Parkinson’s disease pathogenesis

After identifying the potential genetic variants, we proceeded to carry out the enrichment analysis. The pathway analysis was performed using the CytoscapeClueGO plug-in (version 2.5.8). The KEGG, Reactome, and WikiPathways databases were selected in order to obtain a complete compilation of signaling pathways. The enrichment analysis was conducted using the two-sided hypergeometric test, using a Bonferroni step-down adjustment. The terms demonstrated a correlation with each other, as evidenced by a κ score of 0.4. The ClueGO plug-in enables the incorporation of Gene Ontology (GO) terminology and KEGG/BioCarta pathways [4]. This plug-in enables the viewing of biochemical pathways and gene ontologies related to the genetic variants under investigation. Furthermore, it allows for the assessment of functional annotations by comparing them between two clusters. The present study employed a plug-in tool to clarify the molecular pathways that are associated with the genetic changes under research and are linked to PD. The protein-protein interaction (PPI) networks were constructed using the STRING v12.0 database, and further modifications were made using Cytoscape v3.9.1. The assessment was performed by examining physical interactions that displayed a significant level of certainty, indicated by a physical score over 0.4 in the STRING database. The Cytoscape plug-in, CytoHubba, was employed to extract a hub network. The creation of this network involved combining subnetworks formed by the use of degree, closeness, and betweenness techniques, employing the technique of intersectional merging. The Venn diagram tool was used to determine the proteins that are shared throughout the subnetworks, including the top 10 nodes, which were ranked using degree, closeness, and betweenness methods [12]. To visualize the candidate genes at GWAS loci, we submitted the putative genes into the PD GWAS Locus Browser (https://pdgenetics.shinyapps.io/GWASBrowser/).

The identification of proteoforms resulting from post-translational modifications was performed by leveraging the UniProt database after conducting PPI analysis [79]. The biomarkers were categorized using the Panther classification system [80]. The expression of hub proteins in brain tissues was assessed using the Human Protein Atlas, accessible at the online address: https://www.proteinatlas.org/. The ChIP-X Enrichment Analysis Version 3 (CHEA3) was employed to identify the transcription factors that may be accountable for the identified genomic alterations [42]. The present algorithm employs data acquired from the ENCODE and ReMap GTEx datasets to detect transcription factors [43]. The creation of the integrated regulatory network was carried out using the software application Cytoscape. The approach entailed the selection of the top 10 transcription factors, which were chosen based on their average rank score. Later on, the MicroRNA Enrichment Turned Network (MIENTURNET) was employed to generate and analyze networks that depict the relationships between microRNA and its target biomarkers [50]. We performed microRNA-target interaction analysis using the MIENTURNET tool, with 310 curated variant-associated genes as input. Enrichment analysis was conducted using KEGG, Reactome, and WikiPathways databases, applying a significance threshold of *p* < 0.05 after Benjamini-Hochberg correction. miRNAs were identified based on its significant enrichment in PD-relevant pathways, a minimum interaction score defined by MIENTURNET defaults, and further validated through brain-specific expression profiles from the Human Protein Atlas and supportive literature evidence. The functional enrichment analysis employed the KEGG, Reactome, and WikiPathway databases in combination with the disease ontology database. A significance level of 0.05 was established in order to identify the functional annotations that exhibited significant enrichment throughout the whole gene group in the input set. The P-values underwent correction using the Benjamini-Hochberg approach [51]. The study utilized the R programming language version 4.0.2 for performing general data transformations and groupings. The activities were completed using the tidyverse package collection (version 1.3.2). The charts were created using the R packages ggplot2 v3.4.0, ggrepel v0.9.3, and ggpubr v0.5.0. The figure panels were created using the online platform BioRender.com.

## Results

**Fig. 1:**
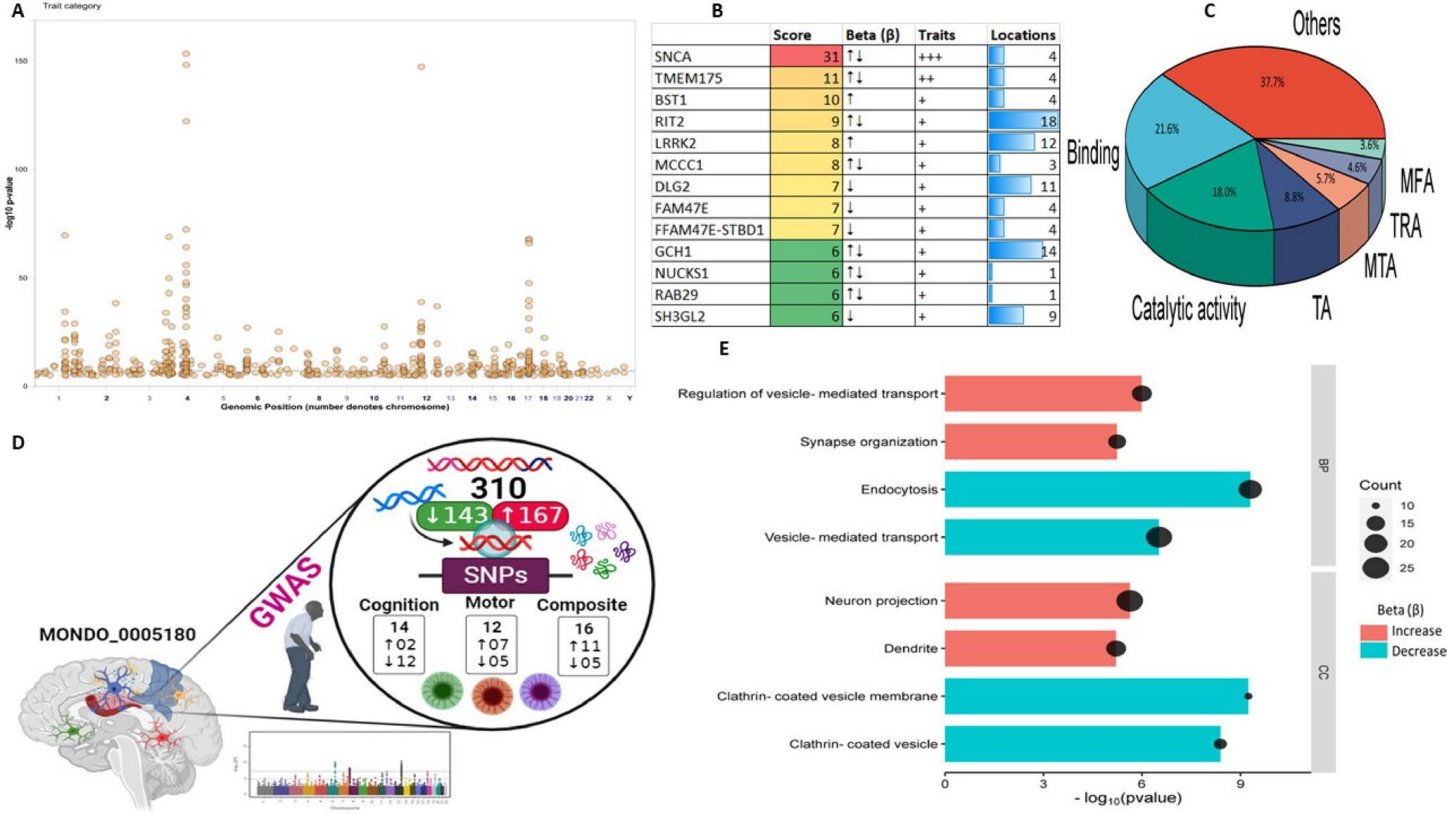
Genetic variants associated with Parkinson’s disease (PD) etiology. Manhattan map of the genome-wide loci linked to Parkinson’s disease (PD) (**A**). Common genetic variants and traits associated with PD etiology (**B**). Genetic variation classification (**C**) and chosen genetic variants linked to PD for further examination (**D**). Biological processes (PP) and cellular components (CC) are included in the top enriched analysis for genetic variants linked to PD (167 biomarkers for high risk and 143 biomarkers for low risk) (**E**). The GWAS database’s biomarker frequencies are used to calculate the score. Transcription regulator activity (TRA), transporter activity (TA), molecular function regulator activity (MFA), and molecular transducer activity (MTA) Others, such as molecular adaptor activity, ATP-dependent activity, antioxidant activity, and structural molecule activity. +++: Age at onset, Parkinson disease, and Parkinson disease progression measurement; ++: Age at onset and Parkinson disease; +: Parkinson disease. Decrease ($\downarrow$): a decreased likelihood of PD (a negative beta value); Increase ($\uparrow$): an increased likelihood of PD (a positive beta value)

This study used data from 68 studies (after cleaning data) about genetic variants implicated in the pathogenesis of PD. A total of 542 variant and risk alleles were located across all chromosomes, especially chromosomes 4 and 17. As shown in Fig. 1A**–**B, *SNCA* and *TMEM175* located in chromosome number 4 showed high frequencies and traits (e.g., age at onset, cognitive progression, motor progression, composite progression, and tremor dominant and postural instability gait difficulty, etc.). The majority of these genes included various variant and risk alleles, which exert doubled nature (increase ($\uparrow$) or decrease ($\downarrow$) risk of PD). Most of these genes exert binding (21.6%) and catalytic activities (18.0%) (Fig. 1C). After the cleaning process, there were 310 genetic variations, including 167 with a higher risk of PD and 143 with a lower risk of PD (Supplementary Table S2). Furthermore, there were 14, 12, and 16 genetic variations associated with the cognitive, motor, and composite progression of PD (Fig. 1D). There was significant evidence of impaired synaptic function, vesicle-mediated transport, and neuron projection associated with variant and risk alleles of PD (Fig. 1E).

**Fig. 2:**
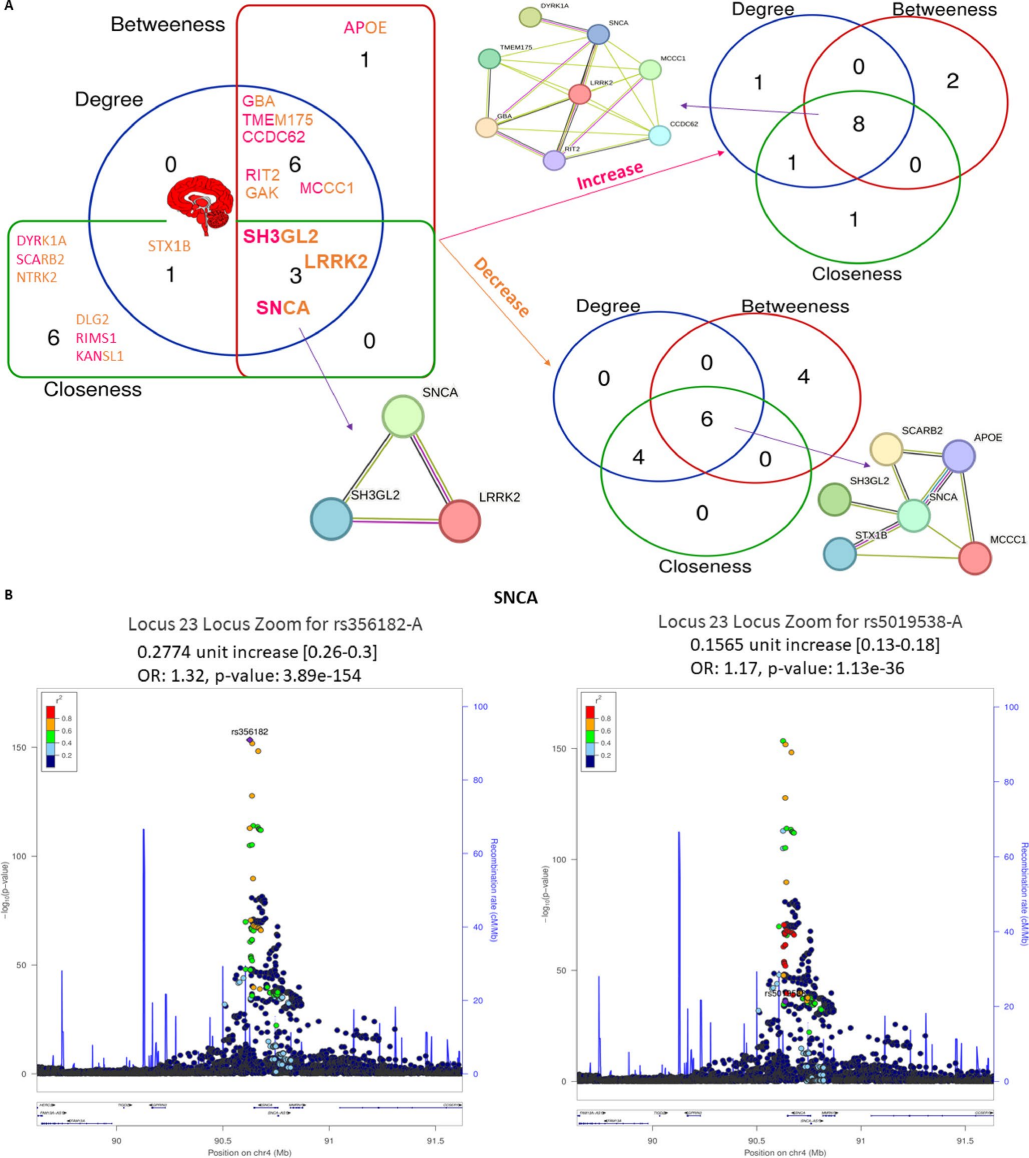

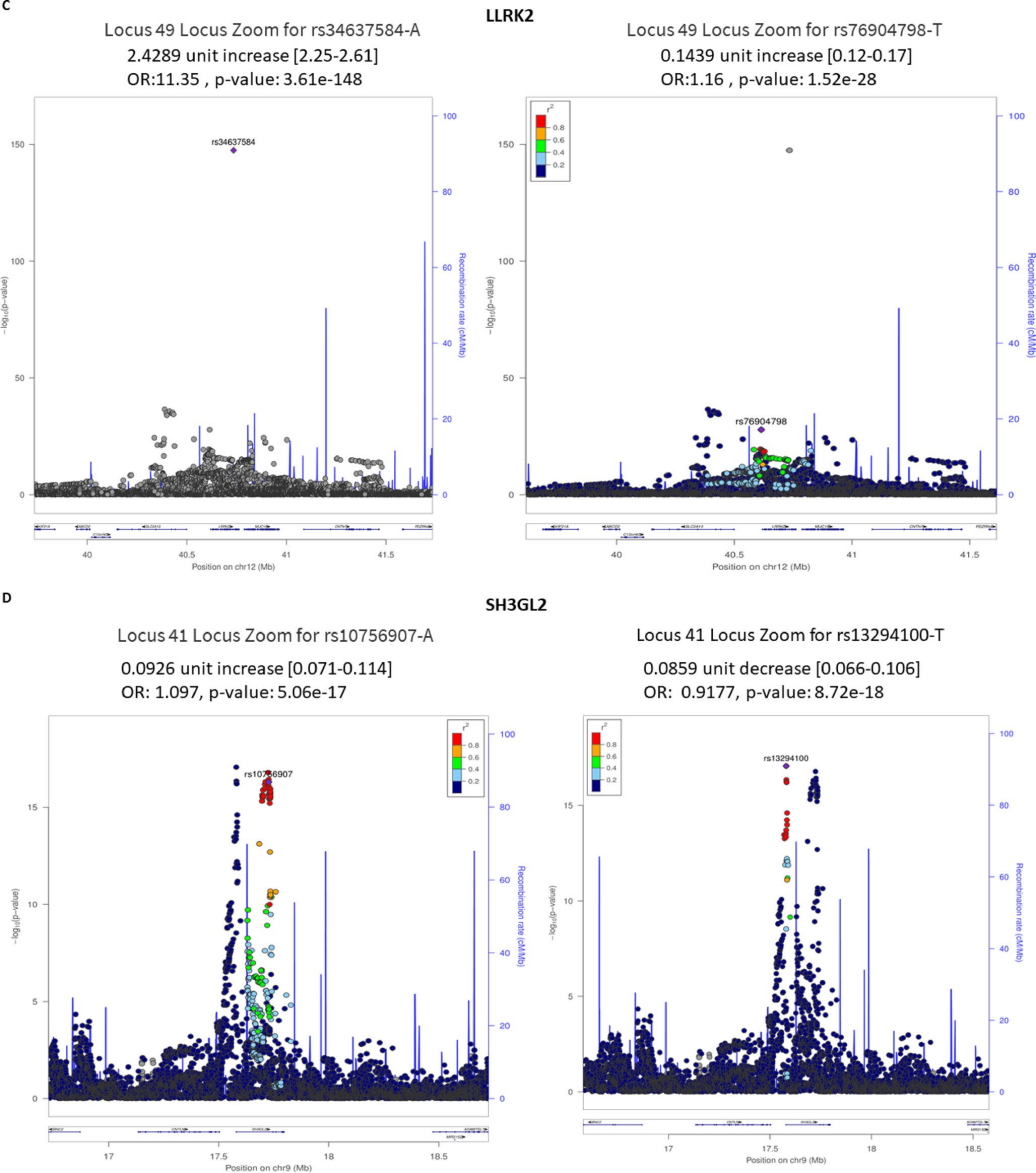
Topological analysis and key gene loci associated with Parkinson’s disease (PD). (**A**) Network topological measures including betweenness, closeness, and degree centrality used to identify hub genetic variants in PD (**B**–**D**) Key loci identified by the PD GWAS Locus Browser: (**B**) SNCA, (**C**) LRRK2, and (**D**) SH3GL2, highlighting important variant positions related to PD risk

**Fig. 3:**
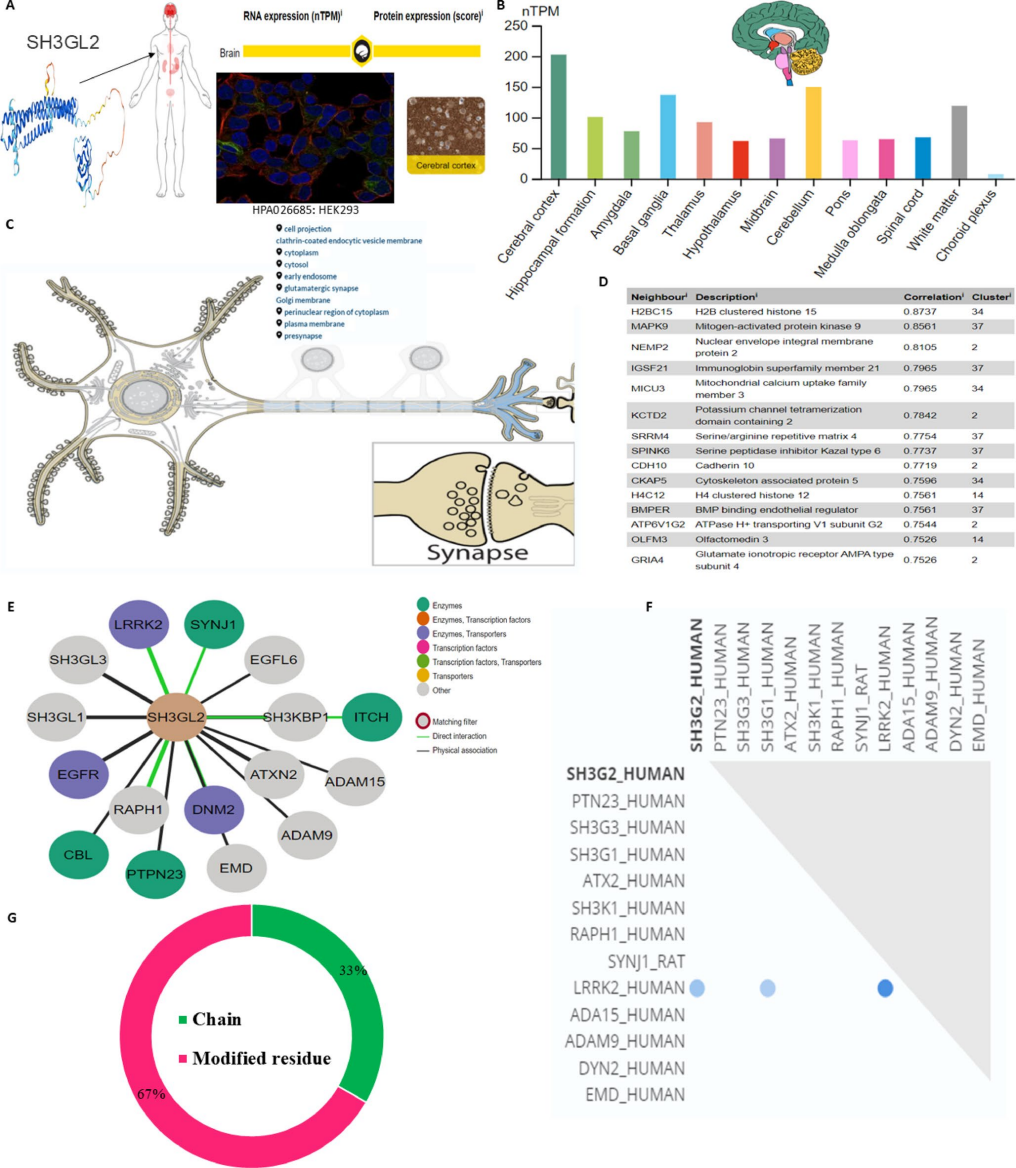
Expression and functional network of SH3GL2 in Parkinson’s disease. (**A**–**B**) 3D-structure of SH3GL2 and expression distribution of SH3GL2: (**A**) in HEK293 cell line, (**B**) across different brain regions, and within neuronal compartments including clathrin-coated endocytic vesicle membrane, glutamatergic synapse, and presynapse (Human Protein Atlas (https://www.proteinatlas.org). The nTPM (transcripts per million) value denotes the number of transcripts detected for the SH3GL2 gene. 3D-structure of SH3HL2 was retrieved from the AlphaFold web tool (https://alphafold.ebi.ac.uk/entry/Q8IZ09). (**C**) Correlation analysis showing SH3GL2 association with H2B clustered histone 15 and mitogen-activated protein kinase 9 within neuronal clusters (obtained from Uniprot (https://www.uniprot.org/uniprotkb/Q99962/entry). (**D**–**F**) Protein-protein interaction (PPI) network of SH3GL2 with enzymes (SYNJ1, CBL, PTPN23, ITCH) and transporters (LRRK2, EGRR, DNM2) in the brain (**G**) Post-translational modifications of NCAM1 linked to PD pathophysiology

**Fig. 4:**
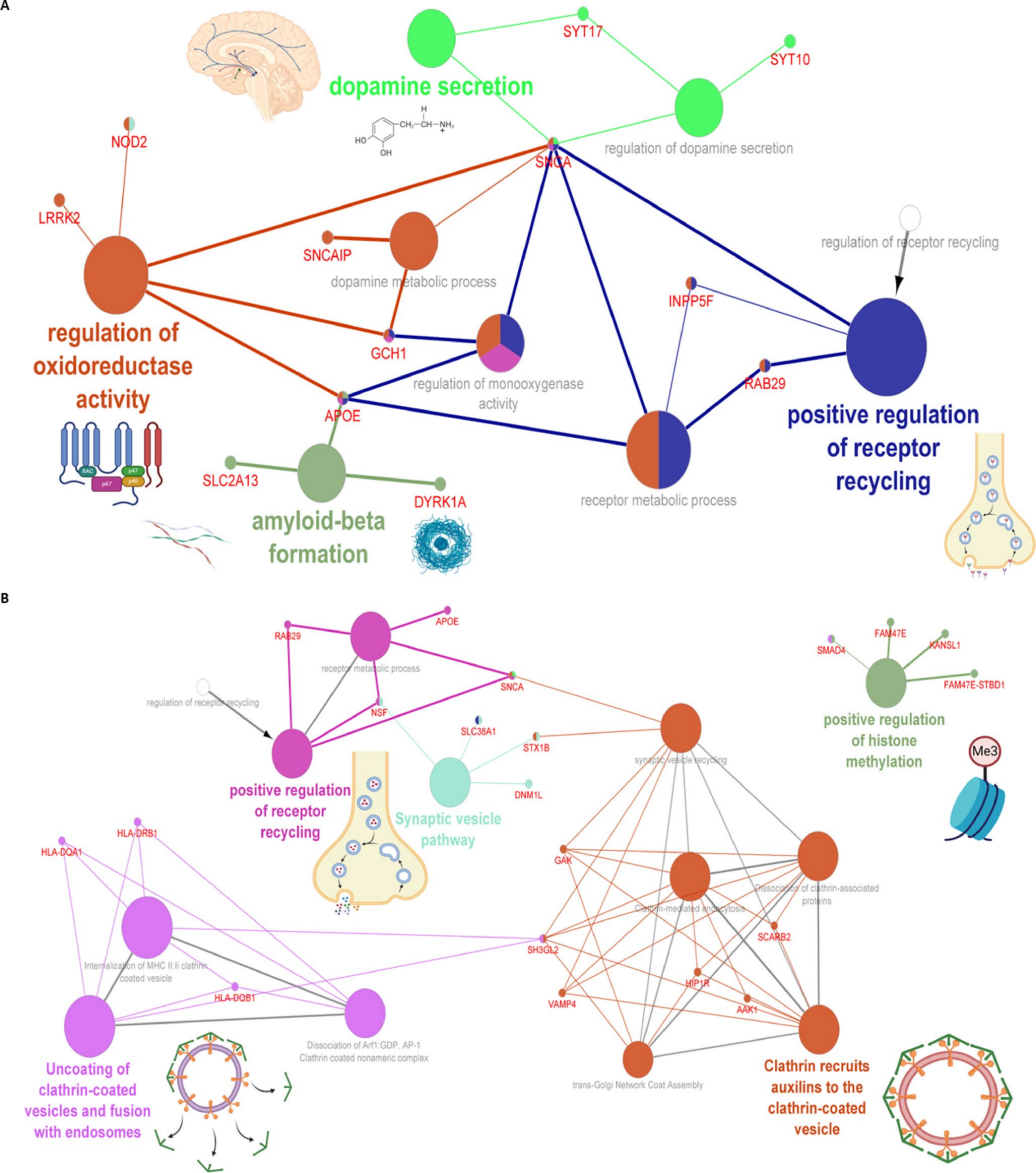

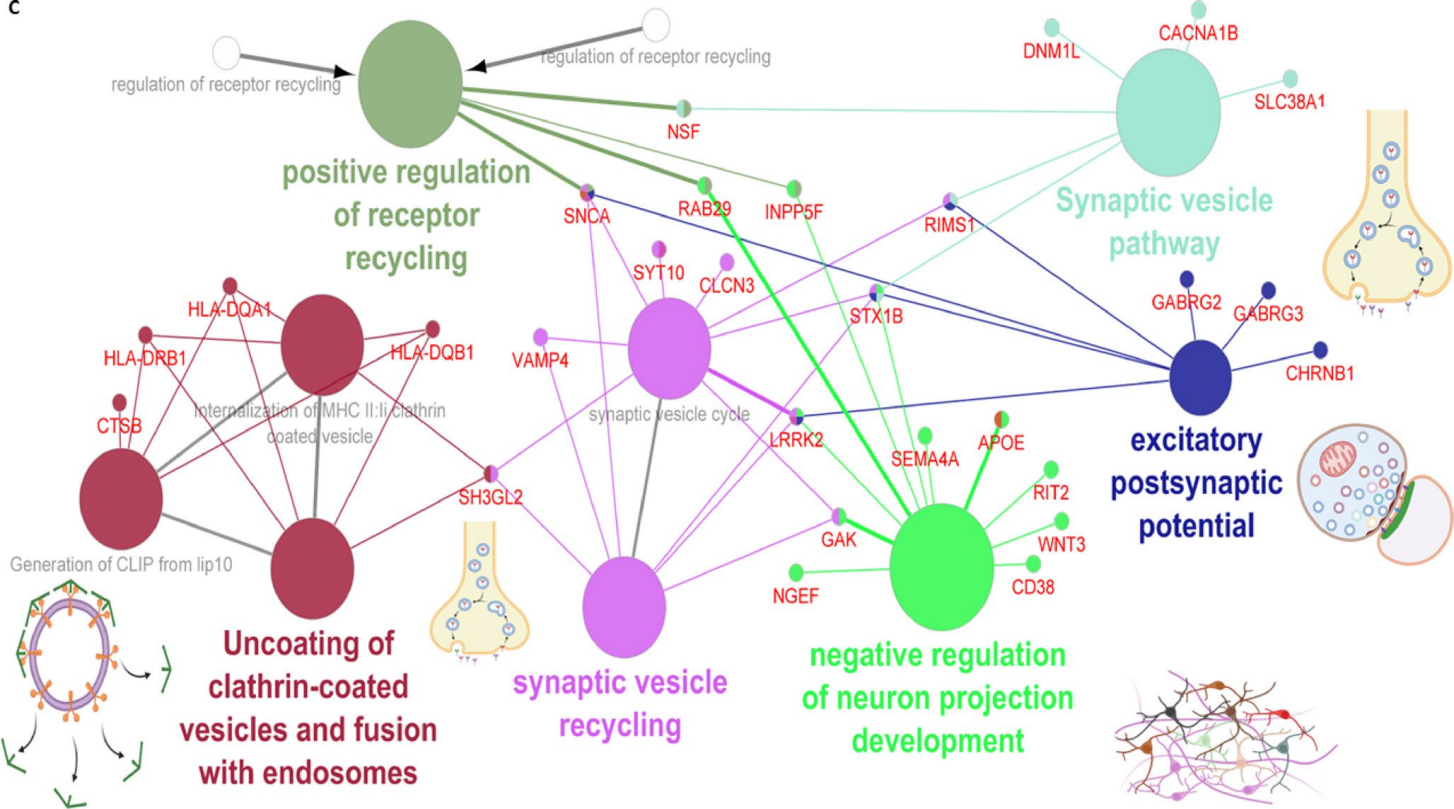
Protein-protein interaction enrichment and genetic variation analyses in Parkinson’s disease. (**A**–**C**) PPI enrichment analysis of genetic variants associated with PD risk: (**A**) 167 high-risk biomarkers, (**B**) 143 low-risk biomarkers, and (**C**) combined set of 310 high- and low-risk biomarkers, demonstrating molecular pathways linked to PD pathogenesis. In the networks, nodes represent genes or pathways, edges represent functional associations or shared interactions, and arrows indicate directional relationships between genes and pathways

Three hub genes were identified after network topological analysis (Fig. 2A–D), including *SNCA *($\uparrow$rs5019538 and $\uparrow$rs356182)*, *(*LRRK2* ($\uparrow$rs34637584 and $\uparrow$rs76904798), and *SH3GL2* ($\uparrow$rs10756907 and $\downarrow$rs13294100). It has been well known that *SNCA* and *LRRK2* are the prevalent genes underlying PD etiology. So, this study only analyzed the expression of *SH3GL2*. As shown in Fig. 3A–B, the expression of *SH3GL2* was mostly in the brain, especially in the cerebral cortex. *SH3GL2* is also found in the clathrin-coated endocytic vesicle membrane, glutamatergic synapse, and presynapse (Fig. 3C). *SH3GL2* was found to be a part of cluster 2 neurons – nucleosome; *SH3GL2* showed high correlations with *H2B* clustered histone 15 (*r* = 0.874, cluster 34) and mitogen-activated protein kinase 9 (*r* = 0.856, cluster 37) (Fig. 3D). We also found *SH3GL2* interacted with other enzymes (*SYNJ1*,* CBL*,* PTPN23*, and *ITCH*), transporters (*LRRK2*,* EGRR*, and *DNM2*), and other proteins in the brain (Fig. 3E–F). There was substantial evidence of impaired dopamine secretion, receptor recycling, and oxidoreductase activity and increased amyloid-beta formation associated with genetic variations with a higher risk of PD (Fig. 3G). Significant evidence indicated improved synaptic vesicle pathway, neuron projection development, and regulated histone methylation and excitatory postsynaptic potential related to genetic variants that carry a lower risk of PD (Fig. 4A–C).

**Fig. 5:**
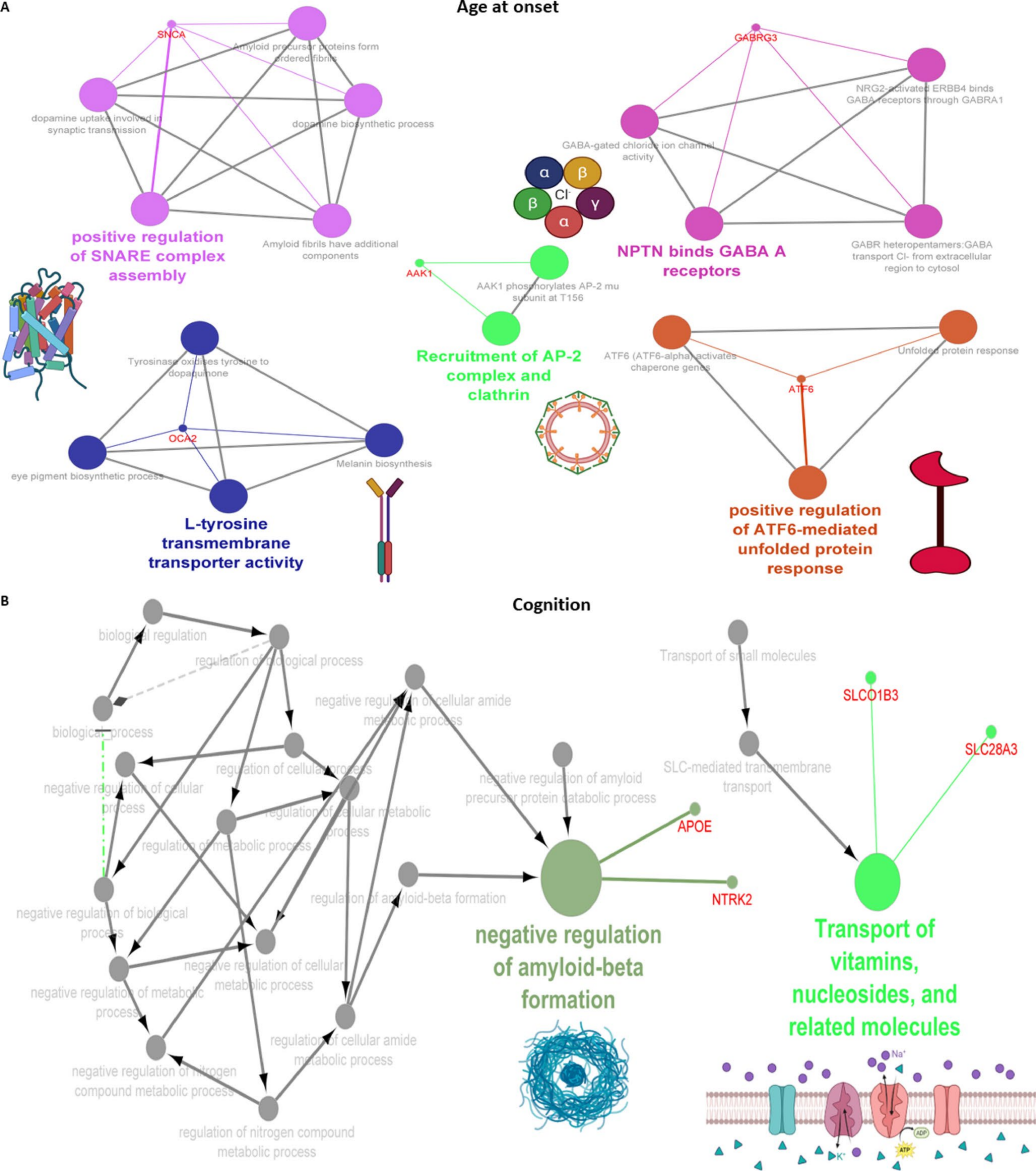

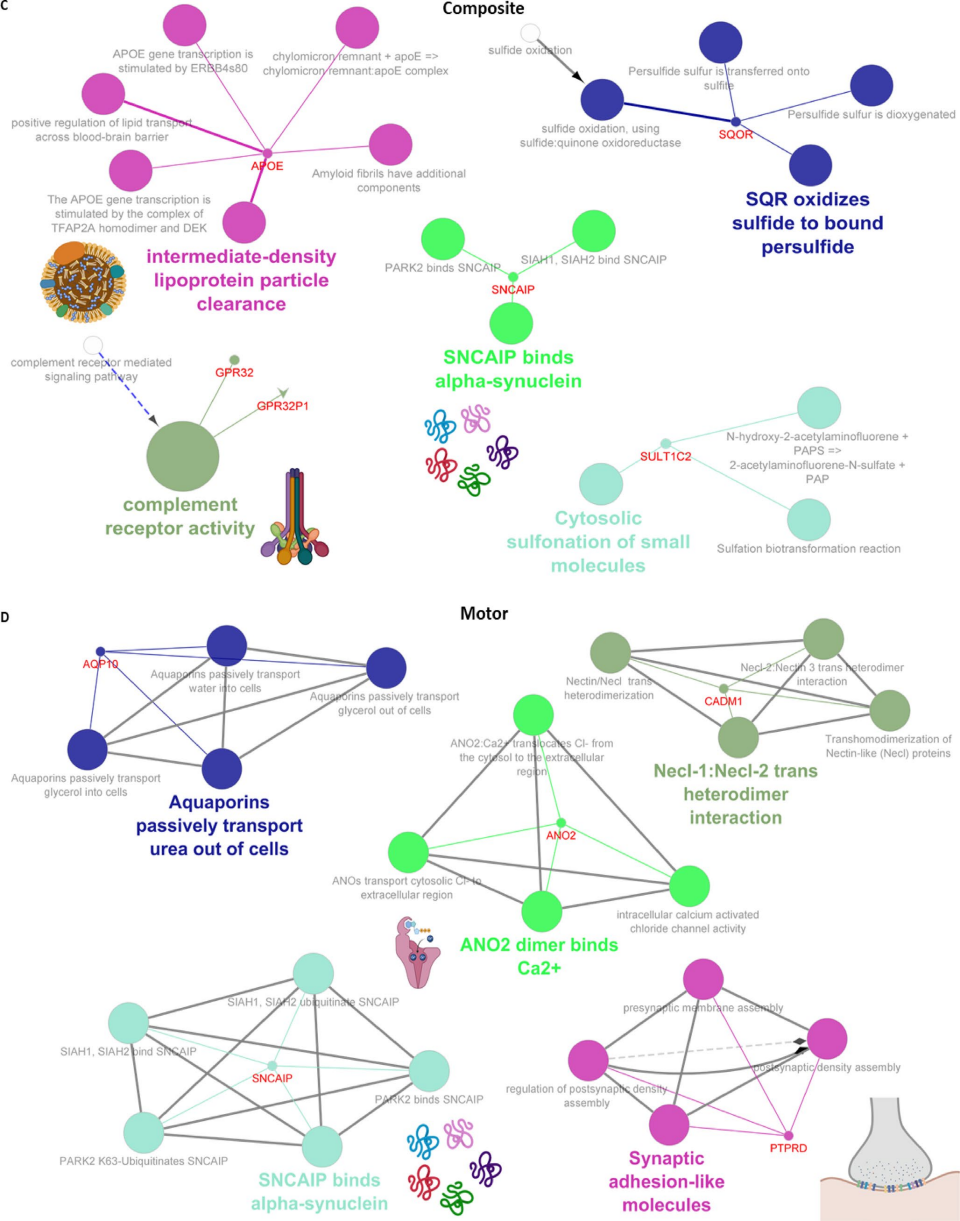

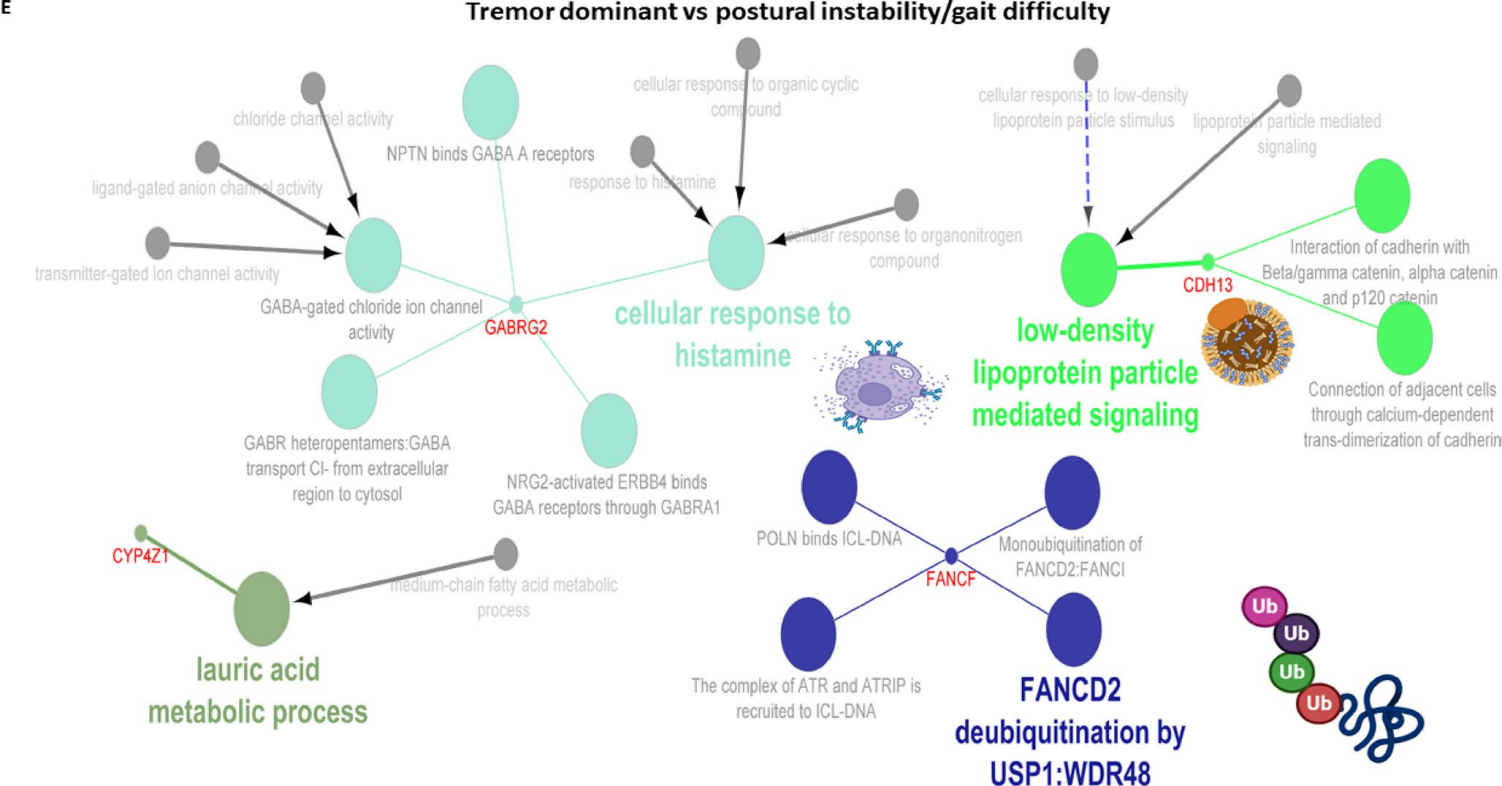
Putative genes and protein-protein interaction (PPI) involved in clinical feature of Parkinson’ disease (PD). PPI enrichment analysis of putative genes involved in age at onset of PD (**A**), PD-associated cognitive impairment (**B**), Progression of motor symptoms in PD (**C**), Progression of composite symptoms in PD (**D**), and tremor dominant and postural instability/gait difficulty (PIGD) phenotypes of PD (**E**). In the networks, nodes represent genes or pathways, edges represent functional associations or shared interactions, dashed lines indicate functional similarity, and arrows indicate directional relationships between genes and pathways

Next, we further analyzed the molecular mechanisms underlying the pathogenesis of the PD subgroup, including age at onset, cognitive progression, motor progression, composite progression, and tremor dominant and postural instability gait difficulty. Genetic variations (*SNCA* ($\uparrow$rs356203), *AAK1* ($\downarrow$rs7577851), *OCA2* ($\downarrow$rs17565841), *GABRG3* ($\downarrow$rs17565841), and *ATF6* ($\downarrow$*rs10918270*)) associated with age at onset of PD were found to be related to *SNARE* (soluble N-ethylmaleimide sensitive factor attachment protein receptor) complex assembly, ly-tyrosine transmembrane transporter activity, recruitment of *AP-2* complex and clathrin, *GABAA* receptors, and *ATF6* (activating transcription factor 6)-mediated unfolded protein response (Fig. 5A). In terms of cognitive progression, genetic variations (*APOE* ($\downarrow$rs429358), (*NTRK2* ($\downarrow$rs148603475), *SLCO1B3* (*SLC28A3* ($\downarrow$rs148603475)) were related to negative regulation of amyloid beta formation and transport of vitamins, nucleosides, and related molecules (Fig. 5B). As shown in Fig. 5C, genetic variations (*AQP10* ($\downarrow$rs35950207), *SNCAIP* ($\uparrow$rs5870994), *ANO2* ($\downarrow$rs74709761), *CADM1* ($\uparrow$rs4436579), and *PTPRD* ($\uparrow$rs7870456)) were underlying the motor progression of PD via altering aquaporins that passively transport urea out of cells, binding to alpha-synuclein and Ca^2+^, *Necl-1:Necl-2* trans heterodimer interaction, and synaptic adhesion (Fig. 5D). Furthermore, we found genetic variations (*APOE* ($\uparrow$rs429358), *GPR32* ($\uparrow$rs4802739), *GPR321* ($\uparrow$rs4802739), *SNCAIP* ($\uparrow$rs17367669), *SQOR* ($\uparrow$rsrs17554587), and *SULT1C2* ($\uparrow$rs13424530)) underlying the composite progression of PD by altering complement receptor activity, lipoprotein particle clearance, binding alpha synuclein, *SQR* (sulfide quinone oxidoreductase), oxidizing sulfide to bound persulfide, and cytosolic sulfonation (Fig. 5E). On the other hand, we also found genetic variations (*GABRG2* ($\uparrow$rs11949046), *CYP4Z1* ($\uparrow$rs116504637), *CDH13* ($\downarrow$rs13330839), *FANCF* ($\downarrow$rs55971529)) were involved in the pathogenesis of tremor dominant and postural instability gait difficulty by altering low-density lipoprotein particle-mediated signaling, cellular response to histamine, lauric acid metabolic process, and *FANCD2* deubiquitination (Fig. 5F).

**Fig. 6:**
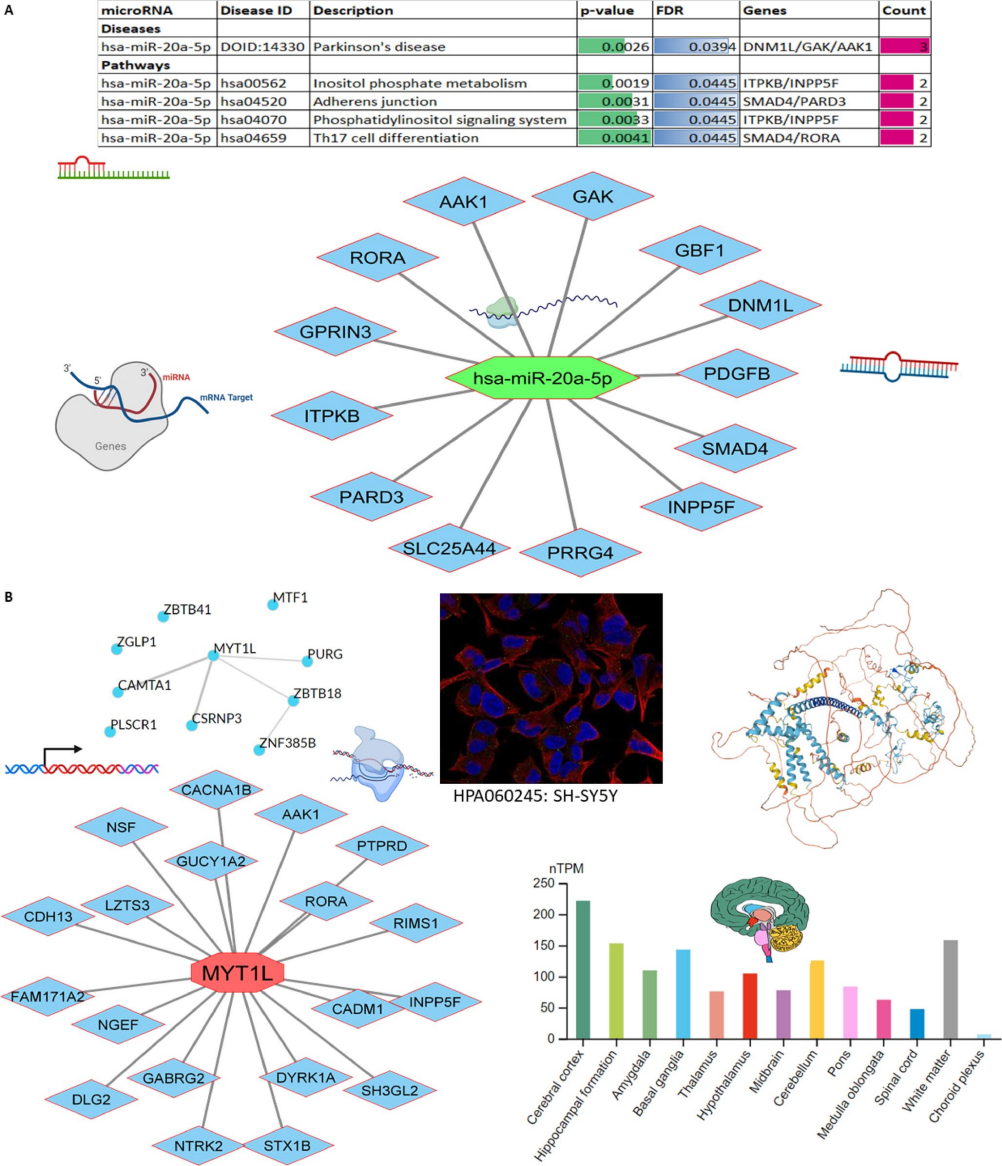
Networks of microRNAs (**A**) and transcription factors (**B**) linked to genetic variants implicated in the etiology of Parkinson’s disease (PD). Using 310 genetic variants associated with PD, networks of miRNAs and transcription factors were evaluated

Most miRNAs are found in the central nervous system, especially during brain development [46]. Dysregulation of miRNA transcript expression has been observed in various neurodegenerative diseases, including PD [75]. We performed microRNA–target interaction analysis using the MIENTURNET tool, with 310 curated variant-associated genes as input. Enrichment was assessed against KEGG, Reactome, and WikiPathways databases, applying a significance threshold of *p* < 0.05 after Benjamini–Hochberg correction. We identified three candidate miRNAs (hsa-miR-16-5p, hsa-miR-17-5p, and hsa-miR-20a-5p); however, only hsa-miR-20a-5p showed significant enrichment (false discovery rate (FDR) = 0.0394) in PD-relevant pathways. Specifically, hsa-miR-20a-5p was associated with inositol phosphate metabolism, adhesion junction, phosphatidylinositol signaling, and Th17 cell differentiation (Fig. 6A, Supplementary Table S3). We also identified the transcription factors underlying the pathogenesis of PD associated with genetic variations. As shown in Fig. 6B, *MYT1L* (myelin transcription factor 1 like) was the predominant transcription factor (the first ranking based on mean-rank) among the top 10 transcription factors. The expression of *MYT1L* was found in the SH-SY5Y cell line and across the brain, especially the cerebral cortex.

## Effect size analysis of genetic variants in Parkinson’s disease

To address the contribution of effect sizes to biomarker identification, we analyzed the beta values of the 232 genetic variants associated with PD and related traits from 68 GWAS studies (Supplementary Table S1). Beta values, representing the change in PD risk or trait progression per risk allele, were categorized by effect direction: 125 variants were risk-increasing (mean beta = 0.245, SD = 0.346, range = 0.0529–2.4289), and 107 were risk-decreasing (mean beta = − 0.209, SD = 0.231, range = − 2.84 to − 0.0529). These statistics highlight the variability in effect sizes, with risk-increasing variants showing larger magnitudes on average, driven by outliers such as *LRRK2* (rs34637584, beta = 2.4289, *p* = 4e-82) and *GBA1* (rs421016, beta = 1.979, *p* = 1e-14). To illustrate the distribution of effect sizes, box plots were generated for risk-increasing and risk-decreasing variants across traits (Supplementary Fig. S1). These plots highlight the variability in beta values and emphasize variants with |beta| >0.3, particularly for *LRRK2*,* GBA1*, and *SNCA*, which are critical for PD pathogenesis.

### Trait-Specific effect sizes

Variants were grouped by clinical traits to assess their impact on PD pathogenesis, including PD risk, age at onset, motor progression, cognitive progression, composite progression, motor subtype, and mortality. PD Risk (177 variants): The largest effect sizes were observed for *LRRK2* (rs34637584, beta = 2.4289), *GBA1* (rs421016, beta = 1.979), and *ASS1P14/SYT10* (rs138895122, beta = 0.6116), indicating strong contributions to PD susceptibility. *SNCA* variants (e.g., rs356182, beta = −0.343; rs5019538, beta = −0.1565) and *TMEM175* (rs34311866, beta = 0.2425) also showed moderate to large effects, aligning with their roles as hub genes in Lewy body formation and lysosomal function, respectively. Age at Onset (6 variants): *OCA2/GABRG3* (rs17565841, beta = −2.84, *p* = 3e-6) and *QSER1/PRRG4* (rs10767971, beta = 3.24, *p* = 5e-7) had the largest effects, associated with earlier and later disease onset, respectively. *SNCA* (rs356203, beta = 1.4337) and *TMEM175* (rs34311866, beta = −0.613) further influence onset timing. Motor Progression (9 variants): *ANO2* (rs74709761, beta = −0.41) and *GPR19* (rs12813102, beta = 0.43) exhibited significant effects, reflecting their roles in motor symptom progression via synaptic and signaling pathways. Cognitive Progression (9 variants): *SLCO1B3* (rs143371462, beta = −0.64) and *APOE* (rs429358, beta = −0.38) showed substantial effects, consistent with their involvement in cognitive decline and amyloid-beta pathology. Composite Progression (8 variants): *FAM184A* (rs79987229, beta = 0.85) and *SQOR* (rs17554587, beta = 0.85) had the largest effects, linked to inflammation and oxidative stress pathways. Motor Subtype (Tremor vs. PIGD, 7 variants): *FANCF* (rs55971529, beta = −1.08) and *TPI1P1* (rs988295487, beta = −0.84) were notable for influencing motor phenotypes through DNA repair and metabolic pathways. Mortality (1 variant): *RPL3/PDGFB* (rs12628329, beta = 1.79) significantly affected survival outcomes.

## Integration with hub genes and pathways

High-effect-size variants were mapped to hub genes identified in our network analysis. *LRRK2* (rs34637584, beta = 2.4289) and *GBA1* (rs421016-G, beta = 1.979) showed the largest effects, reinforcing their roles in autophagy and lysosomal dysfunction. *SNCA* variants (e.g., rs356203, beta = 1.4337) underscored its centrality in Lewy body pathology. *TMEM175* (rs34311866-C, beta = −0.613) and *SH3GL2* (rs10756907, beta = −0.0926) aligned with lysosomal and synaptic vesicle pathways, respectively. *BST1* (rs4698412, beta = 0.1035) and *RIT2* (rs12456492, beta = −0.0983) showed moderate effects, consistent with their roles in neuroinflammation and dopamine signaling. *MCCC1* (rs10513789, beta = 0.173) was linked to oxidative stress pathways. These findings confirm that variants with large effect sizes correspond to biologically relevant pathways, enhancing the reliability of our biomarker prioritization.

## Discussion

This study further analyzed the molecular mechanisms underlying the pathogenesis of PD associated with genetic variations. We observed three hub genes (*SNCA*,* LRRK2*, and *SH3GL2*) that carry different variants underlying the pathogenesis of PD. Other biomarkers (*APOE*,* NTRK2*,* SLCO1B3*,* SLC28A3*,* AQP10*,* SNCAIP*,* ANO2*,* CADM1*,* PTPRD*,* GPR32*,* GPR321*,* SQOR*,* SULT1C2*,* GABRG2*,* CYP4Z1*,* CDH13*, and *FANCF*) were also related to the clinical characteristics of PD (e.g., cognition, motor, age at onset, etc.). There was significant evidence of altered synaptic function and neuron projection development associated with the studied genetic variations.

### Important genetic variations implicated in the pathogenesis of PD

*SCNA* (alpha synuclein) and *LRRK2* (leucine-rich repeat kinase 2) are well-known biomarkers underlying the etiology of PD. *SNCA* is responsible for encoding the alpha-synuclein protein, which serves as the primary constituent of Lewy bodies (LBs) [73]. The abnormal accumulation of alpha-synuclein plays a critical role in the molecular development of PD, as it results in the production of LBs and other toxic aggregates [24]. Elevated alpha-synuclein production is associated with increased severity in both familiar and sporadic PD patients as a result of the SNCA mutation [34, 66]. Another type of *SNCA*,* SNCAIP* (alpha-synuclein-interacting protein) gene, is also a well-known factor in PD. A genome-wide DNA methylation profiling of 12 PD patients and 12 controls found the expression of *SNCAIP* (alpha-synuclein-interacting protein) gene in the brains of PD patients [15]. Another study also found four missenses (*T383N*,* R606Q*,* N906H*, and *E709Q*) of *SNCAIP* in 202 South African PD patients [44]. *APOE* gene encodes a protein underlying lipid metabolism. Various *APOE* alleles have distinct impacts on the progression of PD and the accumulation of central amyloidopathy, specially, *AOPE-ε4* allele, which has a particularly harmful effect. Animals carrying *APOE-ε4* allele demonstrated the most severe alpha-synuclein disease and experienced the shortest lifespan [16, 90]. *APOE-ε4* allele is well known as a key determinant of Alzheimer’s disease and cognitive impairment [38]. In this study, we also observed four variants of *SNCAIP* ($\uparrow$rs5870994), *SNCAIP* ($\uparrow$rs17367669), *APOE* ($\downarrow$rs429358), and *APOE* ($\uparrow$rs429358) involved in the progression of PD, especially in patients with cognitive decline. On the other hand, mutations in *LRRK2* can cause abnormally increased kinase activity, causing PD development [68]. Patients with autosomal dominant PD and those with apparent sporadic PD, who cannot be clinically differentiated from those with idiopathic PD, exhibit *LRRK2* mutations, with the most prevalent mutation being Gly2019Ser [81]. The link between prevalent *SNCA* and *LRRK2* variants and PD has been well described in previous literature [47, 64, 69, 71]. Thus, it is necessary to further analyze the specific variants of *SNCA*,* SNCAIP*,* APOE*, and *LRRK2* for PD management.

Endophilin-A1 is involved in the process of synaptic vesicle endocytosis. Endophilin-A1 is essential for the growth of dendrites, which is dependent on brain-derived neurotrophic factor (*BDNF*). Endophiline-A1 and *SH3GL2* collaborate to facilitate the signaling of *BDNF*- neurotrophic tyrosine kinase receptor 2 from early endosomes and early endocytic trafficking [82]. *SH3GL2* (SH3-domain GRB2-like 2, a synaptic endocytic gene) was discovered in a GWAS meta-analysis of PD, establishing a connection between PD and *SH3GL2* that has a role in regulating synaptic vesicle endocytosis [11]. A new mutation that increases the risk of PD in *SH3GL2* (endophiline-A1) was found, which can impair the protein’s ability to sense calcium, making it immobile and unable to react to calcium influx, which prevents autophagy induction at synapses [17]. Further, a model of *SH3GL2* knockout mice also consistently displayed synaptic endocytic abnormalities, emphasizing the critical need for precise synaptic vesicle endocytosis regulation in preserving the integrity of axon terminals [55]. Adaptor-associated kinase 1 (*AAK1*) is a Ser/Thr protein kinase that plays a crucial role in regulating clathrin-mediated endocytosis [13]. *AAK1* is found throughout the central nervous system and is a crucial factor in the onset age of PD [48]. This study observed that two variants in the hub SH3GL2 gene (($\uparrow$rs10756907 and $\downarrow$rs13294100) and one variant in *AAK1* gene ($\downarrow$rs7577851) contributed to disruptions in synaptic vesicle endocytosis, which play a substantial role in PD pathogenesis. Dysregulation of synaptic vesicle and synaptic adhesion pathways were also listed as the predominant pathways underlying the pathogenesis of PD. These findings suggest that targeting the *SH3GL2* gene as well as other synaptic endocytic genes (*AAK1*) is a promising approach for PD management.

### Other genetic variations implicated in the pathogenesis of PD

*TMEM175* encodes a lysosomal potassium channel critical for regulating lysosomal pH and membrane potential. Alterations in *TMEM175* function disrupt autophagic flux and lysosomal degradation, resulting in the buildup of misfolded proteins like α-synuclein, a key feature of PD pathology [32]. Studies have linked *TMEM175* variants to decreased lysosomal efficiency and heightened neuronal susceptibility in PD models [39]. *BST1* (bone marrow stromal cell antigen 1) plays a role in modulating immune responses and neuroinflammation [36]. Variants in *BST1* are associated with an elevated risk of PD, likely by enhancing chronic microglial activation and inflammatory pathways in the brain [87]. These observations underscore the increasing evidence implicating neuroinflammatory mechanisms in PD progression.

Given that the loss of dopaminergic neurons is the defining characteristic of PD, genes that are specifically expressed in these neurons are potential factors that may contribute to the genetic cause of PD. *SLCO1B3* (solute carrier family 6 member 3) is a human dopamine transporter gene. The association between impaired *SLCO1B3* function and PD has been reported. For example, a meta-analysis study found that the 10-repeat allele of the 40-base pair variable number tandem repeat, a widely investigated genetic variation in the 3’untranslated region of *SLC6A3*, provides neuroprotection in East Asian populations (odd ratio: 0.78; 95%CI: 0.65–0.94), while the presence of the GG genotype and the G allele of the promoter single nucleotide polymorphism *rs2652510* is linked to an increased risk in Caucasians. The allelic G has an odds ratio of 1.26 (95%CI: 1.04–1.54) and genotypic GG has an odds ratio of 1.37 (95% CI: 1.03–1.84) [89]. *GABA* (gamma-aminobutyric acid) exerts a regulatory influence on the pathogenesis of PD that is not influenced by dopaminergic treatment [62, 83]. Dopamine can directly regulate recombinant GABAARs by binding to the β3 subunit, even in the absence of *GABA* [29]. The GABAAR/Cl^−^, HCO3^−^ATPase found in the rat brain plays a role in the phenol-induced symptoms of head-twitching and tremors [54]. A correlation between dopamine and *GABA* was also found in the basal ganglia in the PD model in mice [56]. *CADM1* (cell adhesion molecule 1) is a multifunctional cell adhesion molecule that has been recognized as a tumor suppressor gene [70]. There was evidence of *CADM1* expression in dopamine neurons in the middle brain, especially in the substantia nigra pars compacta, the ventral tegmental area, in the PD patients and a mouse model of peripheral myelinated axons [1, 60]. In this study, various variants were implicated in the processes of dopamine transporters and release, cell adhesion, and receptor recycling, including *SLCO1B3* ($\downarrow$rs143371462), *GABRG2* ($\uparrow$rs11949046), *CADM1* ($\uparrow$rs4436579), and *GABRG3* ($\downarrow$ rs17565841). Therefore, focusing on these genetic variants is crucial in the management of PD.

*ATF6* (activating transcription factor 6) is a type 2 transmembrane protein that is linked with the endoplasmic reticulum (ER). It has a stress-sensing domain in its carboxy-terminal luminal region and functions as a transcription factor with a basic leucine zipper domain [28]. *ATF6*, a defensive component of the unfolded protein response, undergoes processing by COPII-mediated ER-Golgi transport after being activated by ER stress. Alpha-synuclein inhibited the processing of *ATF6* by directly interacting with alpha-synuclein and indirectly by limiting its inclusion in COPII vesicles [78]. *ATF6* signaling dysfunction was accompanied by reduced ER-associated degradation capacity and heightened pro-apoptotic signaling. The inhibition of ATF6 signaling by alpha-synuclein elucidates the involvement of ER stress and the unfolded protein response in PD [14]. This study also highlighted the role of *ATF6* ($\downarrow$rs10918270)), so the elucidation of the inhibitory mechanism of alpha-synuclein on *ATF6* signaling enhances our comprehension of the involvement of ER stress and the unfolded protein response in PD.

The maintenance of water balance in the brain is crucial from both a physiological and therapeutic perspective. Neuronal activity and the regulation of water and ion balance are closely interconnected. Aquaporin, a type of water channel protein, serves crucial roles in facilitating water transport throughout the brain [3]. Changes in levels of *AQP1* and *AQP4* expression were associated with the accumulation of amyloid beta in the brain of people with Alzheimer’s disease and PD [30, 31]. *AQP10* (aquaporin 10) belongs to the aquaglyceroporin family of integral membrane proteins. *AQP10* has been demonstrated to act as a channel that selectively allows the passage of water while also being able to allow the passage of neutral solutes (urea and glycerol) [27]. A GWAS study of 3364 PD patients (mean follow-up of 4.2 years) observed an association between *AQP10*, rs35950207, and motor progression [77]. *ANO2* (anoctamin 2) is a member of a group of chloride channels that are activated by calcium. *ANO2* has a crucial role in various cellular processes, such as the enhancement of olfactory signal transmission and the regulation of neuronal excitability [26]. *ANO2* has been associated with multiple sclerosis, type 3 von Willebrand disease, and Alzheimer’s disease [2, 9, 84]. On the other hand, nucleoside transporters are integral membrane proteins that play a vital role in the process of nucleoside salvage. *SLC28A3* (solute carrier family 28 member 3) is particularly important in this process due to the wider range of substances it can transport and its greater ability to concentrate these substances compared to its members (*hCNT1* and *hCNT2*) [20]. The expression levels of *SLC28A3* were significantly increased in the SH-SY5Y cell PD model caused by 6-hydroxydopamine [49]. This analysis emphasized the role of transporter and water channel underlying the etiology of PD associated with two important genetic variations (*SLC28A3* ($\downarrow$rs148603475), *ANO2* ($\downarrow$rs74709761), and *AQP10* ($\downarrow$rs35950207)).

Dopamine neurons exhibit high levels of the protein tyrosine phosphatase receptor type D (*PTPRD*), which likely contributes to the restructuring of brain networks by influencing the interaction between G protein-coupled receptor hormones and heteromers that regulate dopaminergic modulation [19]. *GPR32* (G protein-coupled receptor 32) participates in the process of macrophage-mediated phagocytosis and the polarization of macrophages towards a pro-resolution phenotype, but it also controls adaptive immune responses by inhibiting the development of T cells into Th1 and Th17 phenotypes and by boosting the production of regulatory T cells [67]. *PTPRD* was found to contribute to the pathogenesis of PD and dopamine-related symptoms (bradykinesia and rigidity) [22]. Neurotrophin has a strong attraction to transmembrane tyrosine kinase proteins known as Trk neurotrophin receptor kinase (*NTRK*) [33]. *BDNF*, a neurotrophin, exhibits a strong affinity for *NTRK2* and significantly influences neural plasticity. *BDNF/NTRK2* also influences the reward circuitry regulated by the dopaminergic circuit [63]. In an in vivo study, the role of the neurotrophin receptor *NTRK2B* in the preservation of dopamine and serotonin neurons in zebrafish was investigated. *GPR32* was implicated in the inflammation processes and PD pathogenesis [10]. This study also found *PTPRD* ($\uparrow$rs7870456), *NTRK2* ($\downarrow$rs148603475), *GPR32* (G protein-coupled receptor 32), and *GPR321* ($\uparrow$rs4802739) underlying the pathogenesis of PD. It is plausible to explain that the dysregulation of the interaction between *PTPRD*,* NTRK2*, and G protein-coupled receptor in dopamine neurons is causing PD development.

*SQOR* (sulfide: quinone oxidoreductase) is an extrinsic membrane protein that facilitates the conversion of sulfide compounds into elemental sulfur through oxidation. Elevating the amount of *SQOR* in the brain, either through adeno-associated virus-mediated gene transfer or sulfide preconditioning by periodically inhaling H2S, suppressed neurodegeneration and enhanced motor dysfunction in PD mice [57]. *SULT1C2* (sulfotransferase family 1 C member 2) is an isoform of the sulfotransferase family implicated in the sulfation of extracellular matrix components. A GWAS study of 856 PD patients observed an association between *ULT1C2* and PD etiology (the Unified Parkinson’s Disease Rating Scale (UPDRS)-IVa-dyskinesia subscore and UPDRS-IVb-fluctuations subscore) [53]. Qualitative analysis and robustness testing of cell type-proportionate changes in PD discovered *SULT1C2*, designating a particular expression in the identified microglia cluster [41]. *CYP4Z1* (cytochrome P450 family 4 subfamily Z member 1) exhibited two single nucleotide polymorphisms that are associated with an odds ratio value greater than 5 in predisposition for PD patients [25]. *CYP4Z1* (rs6675902) was found to be associated with the age-at-onset of PD [61]. The ubiquitous expression of *CYP4Z1* was found in a model of PD in Drosophila [35]. In a Parkinson’s progression markers initiative cohort, *CYP4Z1* exhibited a total of 781 SNPs, with only a small number showing a significantly increased occurrence in PD patients compared to healthy controls [25]. This highlights the significant role of *CYP4Z1* in the etiology of PD. These studies also found three variants *SQOR*, ($\uparrow$rs17554587), *CYP4Z1* ($\uparrow$rs116504637), *SULT1C2* ($\uparrow$rs13424530) underlying the PD etiology. Taken together, SQOR is an enzyme that participates in the process of removing hydrogen sulfide from the body, whereas *SULT1C2* and *CYP4Z1* are a sulfotransferases and *CYP450* enzymes that play a role in the metabolism of many substances, such as hormones and medications. Therefore, the targeting of these enzymes is crucial in the management of PD.

*CDH13* (cadherin-13) facilitates brain plasticity and promotes neuronal development. Genetic mutations in the gene can impair the protein’s ability to inhibit the growth of axons during development and its capacity to protect against oxidative stress [65]. Ultimately, these mutations may contribute to the gradual loss of cells in PD. So, this possibility for PD is intriguing since *CDH13* promotes the movement, growth, and multiplication of neuronal cells [65, 76]. Remarkably, *CDH13* is found in brain areas that are impacted by PD. *FANCF* (Fanconi anemia complementation group F) participants in the development of Fanconi anemia, a study of the serum of idiopathic PD (IPD) patients found lower levels of *FANCF* in the IPD group [88]. *OCA2* (oculocutaneous albinism II) is linked to the typical diversity in eye, skin, and hair color [74]. Increased occurrences of *OCA2* variations have been observed in cases of PD, and there appears to be a connection between *OCA2* and the earlier beginning of PD, suggesting that there may be a mechanism connected to neuromelanin that is responsible for this effect [48]. In this study, we observed that *CDH13* ($\downarrow$rs13330839), *FANCF* ($\downarrow$rs55971529), and *OCA2* ($\downarrow$rs17565841) also play an important role in PD etiology. The genes *CDH13*,* FANCF*, and *OCA2* are not usually acknowledged as significant contributors to the development of PD; more work is needed to elucidate the link between these genes and PD.

On the other hand, this study also found that RIT2 and MCCC1 play an important role in PD pathology. RIT2, a member of the Ras-like GTPase family, has been implicated in the regulation of neuronal signaling and dopamine transporter function, suggesting that variants in this gene may influence dopaminergic neuron vulnerability in PD [23]. MCCC1 encodes a mitochondrial enzyme involved in leucine catabolism, and its dysfunction could contribute to metabolic stress and mitochondrial impairment observed in PD [72]. Together, these genes highlight additional molecular pathways—beyond the classic SNCA and LRRK2 networks—that may modulate disease onset and progression.

### MiRNAs implicated in the pathogenesis of PD

Transcription factors and miRNAs are two important categories of gene expression regulators. Transcription factors regulate gene expression by binding to specific regions of the DNA called promotor regions, whereas miRNAs regulate gene expression after transcription has occurred by connecting with specific regions on the mRNA called 3’ untranslated regions. Disruption of regulatory gene expression can lead to PD [59]. *miR-20a-5p* has a role in causing inflammation in the brain and the buildup of harmful molecules due to oxidative stress. *miR-20a-5p* mitigated mitochondrial dysfunction, inflammation, and cell death caused by the 1-methyl-4-phenyl pyridine ion (MPP+) in HT22 cells by suppressing the *IRF9/NF-κB* axis, which is an in vitro model of PD [85]. *MYT1L* (a transcription factor similar to myelin) has been discovered to have a connection with induced dopaminergic neurons [86]. *MYT1L* demonstrates functioning dopaminergic neurotransmission and alleviates locomotor symptoms in a PD model animal [5], suggesting that it presents novel possibilities for studying transplantation and modeling diseases associated with PD. In line with that, this study also observed an association between *hsa-miR-20a-5p*,* MYT1L*, and PD. These findings suggest that *hsa-miR-20a-5p* and *MYT1L* can be promising targets for developing new treatments for PD.

This study utilized a dataset consisting of 68 previous GWASs to gain a deeper comprehension of the molecular mechanisms underlying gene alterations in individuals with PD. These findings establish the foundation for future inquiries into potential biomarkers for the diagnosis and treatment of PD. Nevertheless, this analysis was dependent on data collected from the GWAS database. Hence, the accuracy and excellence of the interactions in this database play a crucial role in transforming the observed outcomes. While the findings on miRNAs and transcription factors align with previous studies, further investigation is required to validate these results.

## Conclusion

The present work entailed a comprehensive examination of data generated from the GWAS database, with the aim of identifying noteworthy genetic differences and elucidating the underlying molecular pathways connected with the etiology of PD arising from genetic variants. Six common biomarkers (*SNCA*,* TMEM175*,* BST1*,* RIT2*,* LRRK2*, and *MCCC1*) associated with PD were detected across all 68 studies. *SNCA* ($\uparrow$rs5019538 and ($\uparrow$rs356182), *LRRK2* ($\uparrow$rs34637584 and ($\uparrow$rs76904798), and *SH3GL2* ($\uparrow$rs10756907 and ($\downarrow$rs13294100) were the predominant biomarkers underlying PD. Other biomarkers (*APOE*,* NTRK2*,* SLCO1B3*,* SLC28A3*,* AQP10*,* SNCAIP*,* ANO2*,* CADM1*,* PTPRD*,* GPR32*,* GPR321*,* SQOR*,* SULT1C2*,* GABRG2*,* CYP4Z1*,* CDH13*, and *FANCF*) have been found underlying the clinical traits of PD, including age at onset, cognitive progression, motor progression, composite progression, and tremor dominant and postural instability gait difficulty. There was substantial evidence of impaired dopamine secretion, receptor recycling, and oxidoreductase activity and increased amyloid-beta formation associated with genetic variations with a higher risk of PD. Significant evidence indicated improved synaptic vesicle pathway, neuron projection development, and regulated histone methylation and excitatory postsynaptic potential related to genetic variants that carry a lower risk of PD. We also identified *hsa-miR-20a-5p* and *MYT1L* that play a crucial role in elucidating the genetic variants associated with PD. The aforementioned discoveries serve as a fundamental basis for potential therapeutic interventions targeting PD, with a particular emphasis on the genetic variations and mechanisms associated with the condition.

## Supplementary Information

Below is the link to the electronic supplementary material.

Supplementary Material 1

Supplementary Material 2

Supplementary Material 3

## Data Availability

No datasets were generated or analysed during the current study.
