# Supplementary figures and images for: Elucidate biomarkers and the molecular pathways associated with genetic variants that contribute to the etiology of Parkinson’s disease
Source: Acta Neurol Belg. 2025 Sep 30;125(6):1621–41. doi: 10.1007/s13760-025-02897-7 (PMC12644238; doi:10.1007/s13760-025-02897-7)

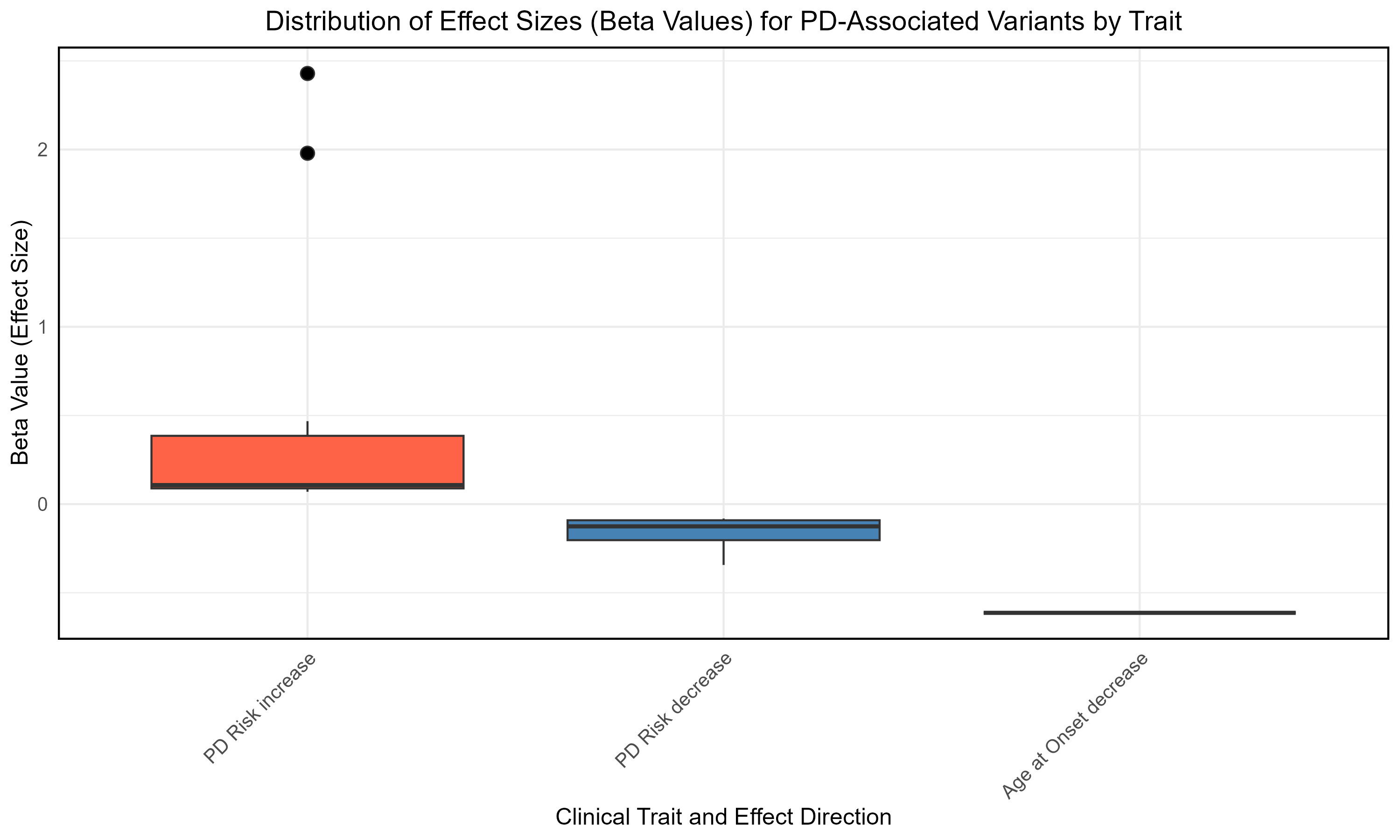

Supplement: Supplementary file 2 — Supplementary Material 2 [file 13760_2025_2897_MOESM2_ESM.png]
