# Supplementary material for: Elucidate biomarkers and the molecular pathways associated with genetic variants that contribute to the etiology of Parkinson’s disease
Source: Acta Neurol Belg. 2025 Sep 30;125(6):1621–41. doi: 10.1007/s13760-025-02897-7 (PMC12644238; doi:10.1007/s13760-025-02897-7)
Supplement: Supplementary file 3 — Supplementary Material 3 [file 13760_2025_2897_MOESM3_ESM.docx]

**Highlight**

- SH3GL2 (⭡rs10756907-A and ⭣rs13294100-T) is a key biomarker for Parkinson’s disease (PD).
- SNCA, TMEM175, BST1, RIT2, LRRK2, and MCCC1 are common genes related to risk alleles and PD.
- Higher-risk PD biomarkers are related to reduced dopamine production
- Biomarkers for PD at lower risk show a notable increase in synaptic functioning.
- hsa-miR-20a-5p and MYT1L are key miRNA and transcription factor.
